# Coronary Vasospasm and Myopericarditis Presenting as ST-Segment Elevation Myocardial Infarction During Immune Checkpoint Inhibitor Therapy

**DOI:** 10.1016/j.jaccas.2026.107924

**Published:** 2026-04-16

**Authors:** Mo'ath Bani Ali, Ahmed Sherif, Emad Hakemi

**Affiliations:** Cleveland Clinic Abu Dhabi, Abu Dhabi, United Arab Emirates

**Keywords:** acute coronary syndrome mimic, coronary vasospasm, hypophysitis, ICI myocarditis

## Abstract

**Background:**

Immune checkpoint inhibitors (ICIs) are associated with rare but potentially fatal immune-related adverse events, including myocarditis, vasculitis, and hypophysitis.

**Case Summary:**

A 53-year-old man with metastatic clear-cell renal cell carcinoma receiving ipilimumab and nivolumab presented with a clinical picture suggestive of acute coronary syndrome. Coronary angiography showed severe triple-vessel disease without a culprit lesion; repeat angiography demonstrated resolution of coronary lesions, consistent with vasospasm. Cardiac magnetic resonance confirmed ICI-associated myocarditis. Concurrent hypophysitis causing secondary adrenal insufficiency was diagnosed. The patient improved with corticosteroids, with full recovery of cardiac function.

**Discussion:**

This case illustrates a rare treatable quadrad of immune-related adverse events—coronary vasospasm, myocarditis, pericarditis, and hypophysitis—and highlights coronary vasospasm as a reversible and underrecognized manifestation of ICI-associated myocarditis.

**Take-Home Messages:**

Coronary vasospasm can mimic obstructive coronary disease and should be considered in immune checkpoint inhibitor myocarditis. Multisystem immune-related adverse events, including myocarditis, pericarditis, and hypophysitis, may present simultaneously and require prompt multidisciplinary care.

## Graphical Abstract

**Figure:**
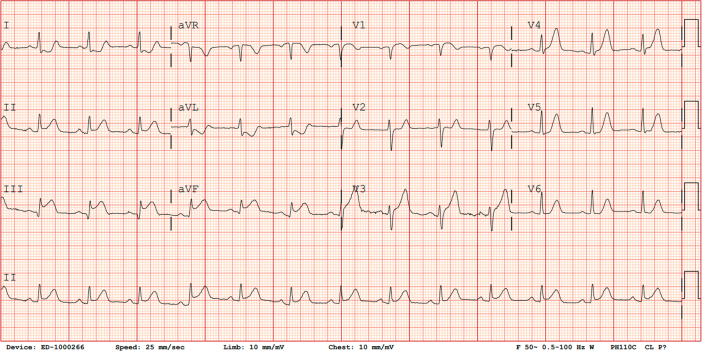

## History of Presentation

A 53-year-old male presented to the emergency department with a 5-day history of chest tightness accompanied by fever, diffuse myalgias, and arthralgias.

**Take-Home Messages**

• Coronary vasospasm can mimic obstructive coronary disease and should be considered in immune checkpoint inhibitors myocarditis.
• Multisystem immune-related adverse events, including myocarditis, pericarditis, and hypophysitis, may present simultaneously and require prompt multidisciplinary care.

## Past Medical History

The patient had a history of metastatic clear-cell renal cell carcinoma (pT3pN0cM1, stage IV). His oncologic course was notable for a left radical nephrectomy and stable bilateral pulmonary metastases on surveillance imaging. He was receiving first-line palliative treatment with combination immune checkpoint inhibitors (ICIs)—ipilimumab and nivolumab—and had completed 5 of 8 planned cycles, with the most recent dose administered 30 days prior to presentation.

## Physical Examination

On arrival, he appeared acutely ill, visibly distressed, and tachypneic. Vital signs revealed a blood pressure of 93/58 mm Hg, heart rate of 90 beats/min, temperature of 38.5 °C, respiratory rate of 22 breaths/min, and oxygen saturation of 95% on room air. His weight was 84 kg, with a body mass index of 24.8 kg/m^2^. Physical examination showed no signs of heart failure. There was no jugular venous distension or peripheral edema, lung fields were clear bilaterally, and heart sounds were normal without murmurs, rubs, or gallops.

## Investigations

Laboratory evaluation revealed a hemoglobin level of 107 g/L, white blood cell count of 10.1 × 10^9^/L, and platelet count of 269 × 10^9^/L. Cardiac biomarkers demonstrated an initial troponin T of 0.3 μg/L, which peaked at 2.7 μg/L on day 2, and N-terminal pro-B-type natriuretic peptide was 265 pg/mL. Creatine kinase was elevated at 1,038 IU/L (normal range 40–200 IU/L), consistent with myocardial injury with possible skeletal muscle myositis. Inflammatory markers were elevated, with a C-reactive protein level of 69.1 mg/L and an erythrocyte sedimentation rate of 82 mm/h. Serum lactate was within normal limits at 0.5 mmol/L. Serum sodium was markedly decreased at 113 mmol/L, and serum creatinine was measured as 106 μmol/L.

**Figure 1:**
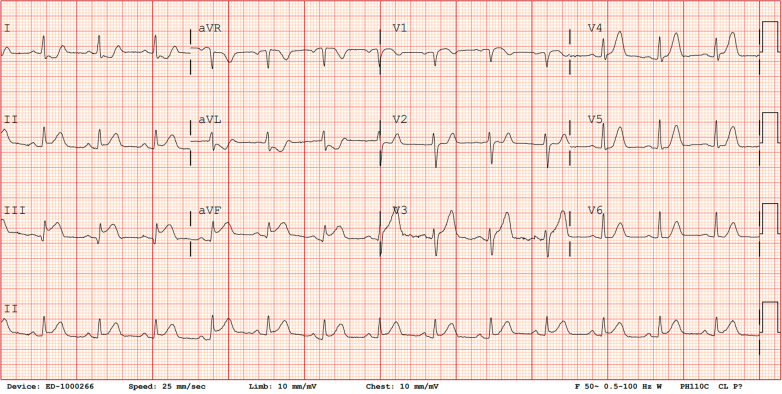
Electrocardiogram The initial electrocardiogram at presentation shows sinus rhythm with a heart rate of 76 beats/min, ST-segment elevation in the inferior leads (II, III, and aVF), and reciprocal ST-segment depression in leads I and aVL.

**Figure 2:**
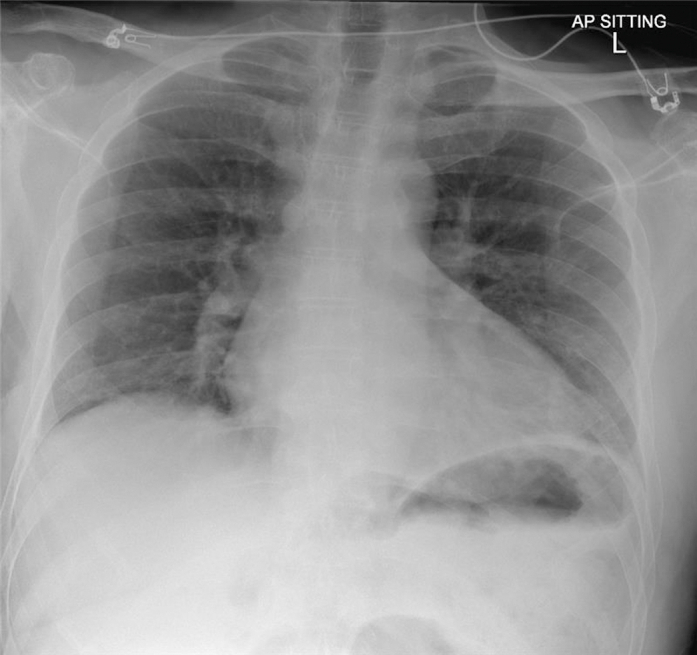
Chest X-Ray Shows underinflated lungs with no evidence of confluent consolidation, pulmonary edema, or pleural effusion.

Electrocardiography (ECG) showed ST-segment elevation in the inferior leads with reciprocal ST-segment depression in leads I and aVL (Figure 1). Chest radiography demonstrated underinflated lungs without evidence of consolidation, pulmonary edema, or pleural effusion (Figure 2). A limited bedside echocardiogram demonstrated preserved ejection fraction (EF) with severe apical hypokinesis.

## Differential Diagnosis

Given the presentation of chest tightness, ECG changes, hemodynamic instability, and elevated cardiac biomarkers, the initial differential diagnoses included acute coronary syndrome, myocarditis, pericarditis, stress-induced cardiomyopathy, and septic shock.

## Initial Management and Subsequent Workup

**Figure 3:**
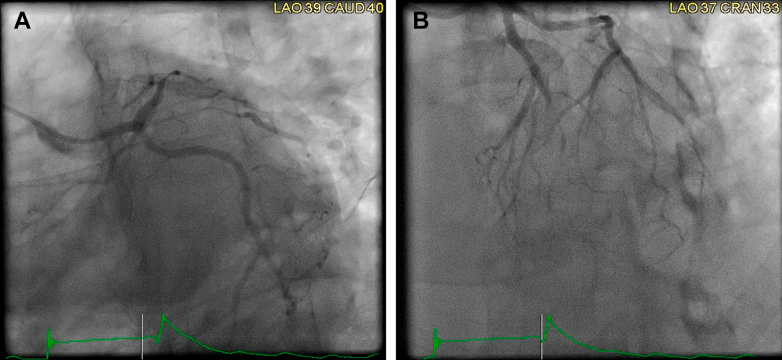
Coronary Angiogram of the Left Coronary System Panel A (LAO caudal view) demonstrates severe ostioproximal stenosis of the ramus intermedius, while Panel B (LAO cranial view) shows severe stenosis of the mid-left anterior descending artery. LAO = left anterior oblique.

**Figure 4:**
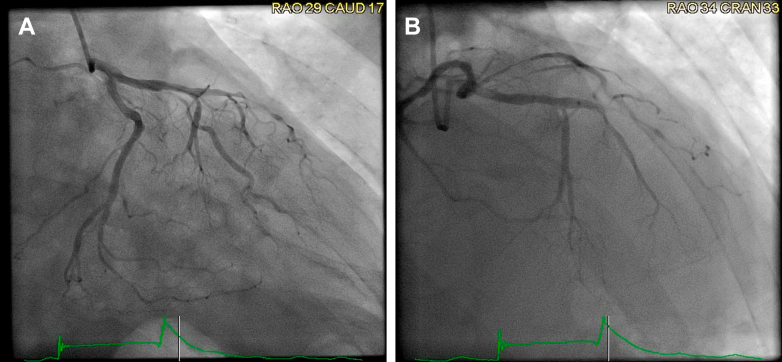
Coronary Angiogram of the Left Coronary System Panel A (RAO caudal view) and Panel B (RAO cranial view) demonstrate severe stenosis of the mid-left anterior descending (LAD) artery. RAO = right anterior oblique.

**Figure 5:**
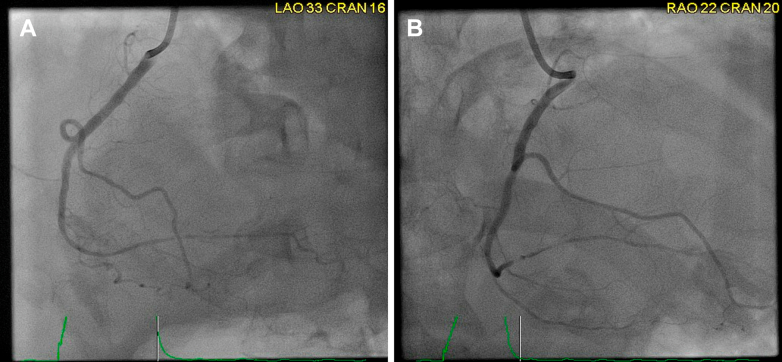
Coronary Angiogram of the Right Coronary System A (left anterior oblique cranial view) and B (right anterior oblique cranial view) demonstrate severe stenosis in the distal right coronary artery (RCA).

In light of the patient's symptoms and the presence of ST-segment elevations on ECG with elevated troponin levels, immediate coronary angiography was performed. It revealed severe triple-vessel disease; severe stenosis in the mid left anterior descending artery, significant stenosis in the ostioproximal ramus, and severe stenosis in the distal right coronary artery, without an identifiable culprit lesion to explain the acute presentation (Figure 3, Figure 4, Figure 5, Video 1, Video 2, Video 3). Nitroglycerin was not administered because of distributive shock and ongoing hemodynamic instability. Given the clinical picture of hemodynamic instability, right-heart catheterization was performed to further evaluate the patient's hemodynamic profile. It revealed normal right atrial and pulmonary capillary wedge pressures, low systemic vascular resistance at 764 dynes s/cm^5^, and normal cardiac output (4.71 L/min) and cardiac index (2.27 L/min/m^2^), consistent with vasodilatory shock. A pulmonary artery catheter was left in place, and the patient was transferred to the intensive care unit for further management. Intravenous norepinephrine was initiated for hemodynamic support, and empiric broad-spectrum antibiotics were started for suspected sepsis.

**Figure 6:**
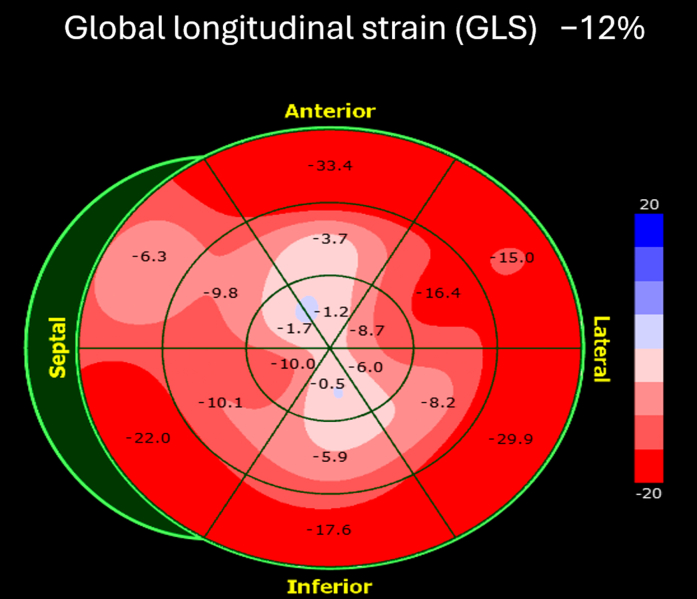
Global Longitudinal Strain Analysis by Speckle-Tracking Echocardiography Speckle-tracking echocardiography demonstrates a reduced global longitudinal strain (GLS) of −12%, with a base-to-apex gradient characterized by markedly reduced strain in the apical segments and relatively preserved basal strain.

Full transthoracic echocardiography done immediately after cardiac catheterization revealed severe hypokinesis of the apical septal, apical inferior, and apical segments, with a preserved left ventricular EF of 55%, a small posterior and lateral pericardial effusion measuring 0.5 cm (Videos 4 and 5), and a reduced global longitudinal strain of −12% (Figure 6).

**Figure 7:**
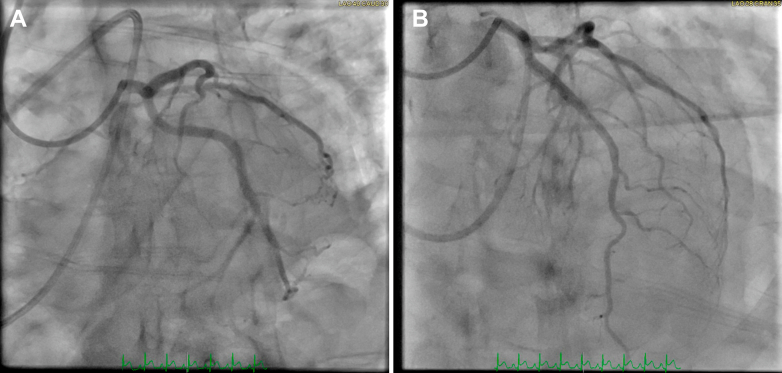
Repeat Coronary Angiogram of the Left Coronary System Panel A (left anterior oblique [LAO] caudal view) shows complete resolution of the stenosis in the ramus intermedius, while Panel B (LAO cranial view) demonstrates complete resolution of the left anterior descending artery stenosis, with normalization of coronary anatomy.

**Figure 8:**
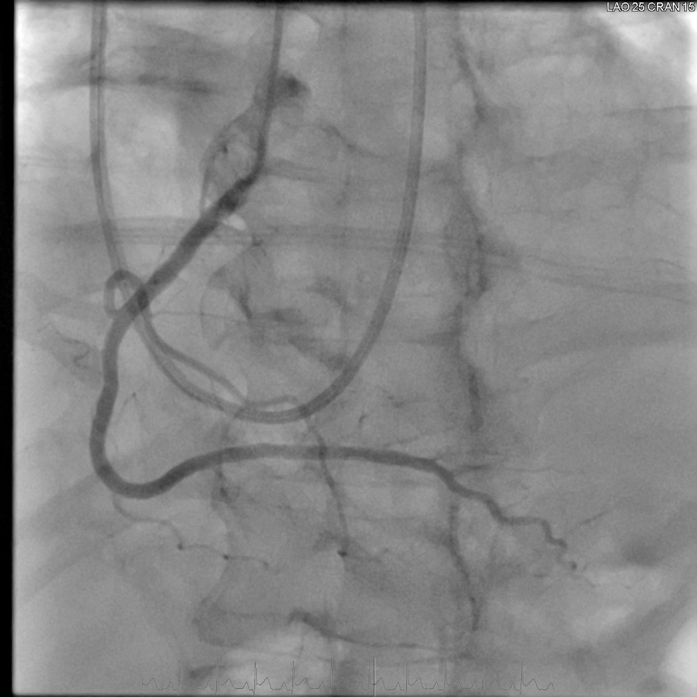
Repeat Coronary Angiogram of the Right Coronary System The left anterior oblique cranial view shows complete resolution of the distal right coronary artery stenosis, with restoration of normal distal blood flow.

Given the upward trend in troponin and ongoing hemodynamic instability, the case was discussed in the multidisciplinary team meeting. Repeat coronary angiography performed the following day for planned revascularization revealed complete resolution of all previously noted coronary stenoses, consistent with transient coronary vasospasm (Figures 7 and 8, Video 6, Video 7, Video 8).

**Figure 9:**
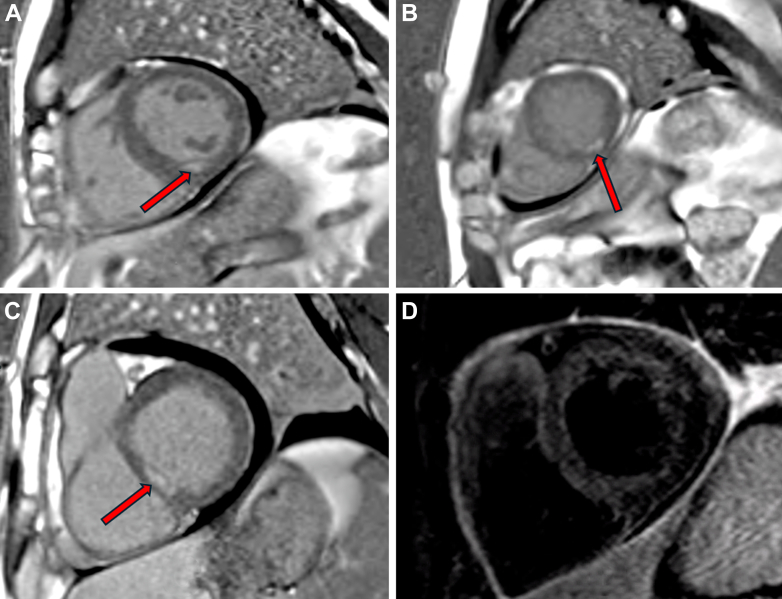
Cardiac Magnetic Resonance Imaging Findings Short-axis late gadolinium enhancement (LGE) images show patchy mid-myocardial enhancement in multiple left ventricular segments (red arrows): (A) mid-inferior wall at the papillary muscle level; (B) apical inferolateral wall; and (C) basal interventricular septum, with a non-subendocardial, noncoronary distribution. (D) Dark-blood T2-weighted short-axis (STIR) imaging shows increased T2 signal in the anteroseptal and anterior walls, consistent with myocardial edema. Combined LGE and T2 findings indicate active myocardial inflammation with associated edema.

Cardiac magnetic resonance imaging demonstrated myocardial edema involving the basal inferoseptal, mid-inferior, and apical segments, along with patchy mid-myocardial late gadolinium enhancement—findings consistent with acute myocarditis (Figure 9).

Further endocrine workup revealed low free thyroxine (T4) at 9.4 pmol/L, low morning cortisol at 23.7 nmol/L, and suppressed adrenocorticotropic hormone at 0.4 pmol/L, consistent with secondary adrenal insufficiency. Thyroid-stimulating hormone was 2.44 mIU/L, and prolactin was mildly elevated at 18.7 mcg/L. Endocrinology consultation confirmed ICI-induced hypophysitis, resulting in secondary adrenal insufficiency and hypothyroidism.

On hospital day 2, following cardiac magnetic resonance imaging confirmation of myocarditis, he was treated with high-dose intravenous methylprednisolone (1 g daily for 3 days), followed by a tapering course of oral corticosteroids. Colchicine was initiated for concomitant pericarditis in view of ST elevations and pericardial effusion, and levothyroxine was also started as guided by the endocrinology team.

## Outcome and Follow-Up

**Figure 10:**
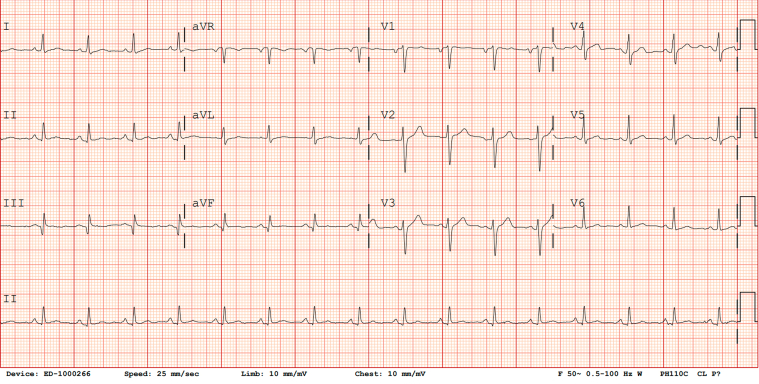
Electrocardiogram Follow-up electrocardiogram after 5 days shows sinus rhythm with a heart rate of 75 beats/min and resolution of the ST-segment elevation in the inferior leads, along with normalization of the reciprocal changes in the lateral leads.

The patient's hemodynamic status stabilized within 72 hours, accompanied by a progressive improvement in troponin levels and inflammatory markers. He was transferred from the intensive care unit to the general medical floor. Antibiotics were discontinued following negative culture results and clinical improvement. ECG changes continued to improve, with near-complete resolution by day 5 (Figure 10). Follow-up transthoracic echocardiography on day 5 demonstrated resolution of the previously observed apical wall motion abnormalities and EF improvement to 60% with persistent small pericardial effusion (Video 9). Troponin levels trended downward, and the patient was discharged on hospital day 7 with a tapering regimen of oral corticosteroids and colchicine. Outpatient follow-up transthoracic echocardiography demonstrated normal left ventricular EF and complete resolution of the pericardial effusion. He remains clinically stable under multidisciplinary follow-up in cardio-oncology, endocrinology, and oncology clinics.

## Discussion

This case illustrates a rare quadrad of immune-related adverse events (irAEs)—coronary vasospasm, myocarditis, pericarditis and hypophysitis—in a patient receiving combination ICI therapy. ICI-associated myocarditis is a potentially fatal but uncommon complication, with an estimated incidence of up to 1.1%, particularly in the setting of combination regimens.^1^, ^2^, ^3^

Emerging data suggest that vascular inflammation and coronary vasospasm may contribute to the cardiovascular toxicity spectrum of ICIs.^2^, ^3^, ^4^ Concurrent ICI-associated myocarditis and vasospastic angina have been recently reported, with symptom resolution following treatment with corticosteroids and coronary vasodilators.^5^ Similarly, in our patient, repeat angiography demonstrated complete spontaneous resolution of triple-vessel stenoses prior to the initiation of immunosuppressive therapy, reinforcing the dynamic nature of the coronary findings. The underlying pathophysiology likely involves immune-mediated endothelial dysfunction and perivascular inflammation, promoting a vasospastic phenotype even in the absence of fixed coronary artery disease.^3^^,^^4^ This challenges traditional assumptions in cardio-oncology and underscores the importance of considering coronary vasospasm in the differential diagnosis of ACS-like presentations in patients undergoing ICI therapy.^2^^,^^6^

Hypophysitis, another irAE observed in this case, is most frequently associated with anti–cytotoxic T-lymphocyte-associated protein 4 agents such as ipilimumab.^7^ Our patient presented with profound hyponatremia, low cortisol, and suppressed adrenocorticotropic hormone levels—findings consistent with secondary adrenal insufficiency. Adrenal dysfunction may exacerbate hemodynamic instability in the context of myocarditis, compounding cardiovascular risk. This highlights the critical role of timely endocrine evaluation and prompt hormone replacement in the management of ICI-related toxicities.^7^^,^^8^

High-dose corticosteroids remain the cornerstone of treatment for ICI-associated myocarditis. Current guidelines from the National Comprehensive Cancer Network and International Cardio-Oncology Society recommend intravenous methylprednisolone 500 to 1,000 mg daily for 3 to 5 days, followed by oral prednisone 1 mg/kg/day with a slow taper over 6 to 12 weeks guided by clinical response and troponin levels.^6^^,^^9^^,^^10^ A systematic review and meta-analysis demonstrated that patients treated with high-dose pulse therapy (1 g/day) exhibited more rapid reduction in myocardial injury biomarkers, lower rates of biomarker rebound, and significantly reduced major adverse cardiovascular events compared to lower steroid doses.^11^ Importantly, high-dose corticosteroids can simultaneously address multiple concurrent irAEs, including myocarditis, pericarditis, and hypophysitis, as observed in our patient.^8^^,^^9^ The National Comprehensive Cancer Network guidelines specifically recommend referral to a tertiary care center for management of complex cases or multisystem irAEs.^9^

The addition of colchicine in this case was guided by the presence of concomitant pericarditis, evidenced by ST-segment elevations and pericardial effusion. While colchicine is well-established for idiopathic pericarditis based on randomized trial data demonstrating a 50% reduction in recurrence, its specific role in ICI-associated pericarditis is less defined.^12^ The International Cardio-Oncology Society position statement recommends that in severe cases of ICI pericarditis with myocardial involvement, treatment with corticosteroids with or without colchicine should be initiated.^10^ Given the myopericarditis phenotype in our patient, the combination of high-dose corticosteroids and colchicine provided comprehensive anti-inflammatory coverage targeting both the T-cell–mediated myocardial inflammation and the pericardial component of disease.

This case highlights several important clinical insights. First, coronary vasospasm can closely mimic obstructive coronary artery disease in ICI myocarditis, and the lesions may be completely reversible. Second, distinguishing dynamic vasospastic lesions from fixed atherosclerotic disease is essential to avoid unnecessary interventions and to guide appropriate immunosuppressive therapy.^1^^,^^2^^,^^5^^,^^6^ Third, early initiation of high-dose corticosteroids is critical, as this single intervention can address multiple concurrent irAEs simultaneously.^9^^,^^11^ Finally, ICI-associated cardiovascular toxicity frequently coexists with other irAEs, such as hypophysitis and pericarditis, necessitating a multidisciplinary approach involving cardio-oncology, endocrinology, and oncology teams.^6^^,^^8^

## Conclusions

Coronary vasospasm should be recognized as a dynamic and reversible manifestation of ICI-associated myocarditis. Repeat angiography with or without intracoronary vasodilatory therapy may be instrumental in differentiating vasospasm from fixed obstructive coronary lesions. Recognition of this entity has important implications for patient care in the evolving field of cardio-oncology.

## Funding Support and Author Disclosures

The authors have reported that they have no relationships relevant to the contents of this paper to disclose.

## Appendix

Video 1 Coronary Angiogram of the Left Coronary System The left anterior oblique caudal view demonstrates severe ostioproximal stenosis of the ramus intermedius.

Video 2 Coronary Angiogram of the Left Coronary System The right anterior oblique caudal view demonstrates severe stenosis of the mid left anterior descending artery.

Video 3 Coronary Angiogram of the Right Coronary System The right anterior oblique cranial view demonstrates severe stenosis in the distal right coronary artery.

Video 4 Echocardiography Apical 4-chamber view shows severe apical hypokinesis with preserved left ventricular ejection fraction (EF) of 55%, a small posterior-lateral pericardial effusion (0.5 cm), normal right ventricle (RV) function, and a pulmonary artery catheter seen in the right atria/RV.

Video 5 Echocardiography With Contrast Apical 4-chamber view focused on the left ventricle, using contrast-enhanced echocardiography with Optison, shows a preserved ejection fraction of 55% with severe apical hypokinesis.

Video 6 Repeat Coronary Angiogram of the Left Coronary System (Left Anterior Oblique Caudal) The left anterior oblique caudal view shows complete resolution of the ostioproximal ramus intermedius stenosis with restoration of normal blood flow.

Video 7 Repeat Coronary Angiogram of the Left Coronary System (Right Anterior Oblique Cranial) The right anterior oblique cranial view demonstrates complete resolution of the left anterior descending artery stenosis, with restoration of normal coronary blood flow.

Video 8 Repeat Coronary Angiogram of the Right Coronary System The left anterior oblique cranial view demonstrates complete resolution of the distal right coronary artery stenosis, with restoration of normal blood flow distally.

Video 9 Repeat Echocardiography The study shows normalization of left ventricular systolic function with an ejection fraction of 64%, complete resolution of apical hypokinesis, and a persistent small pericardial effusion.
